# (2*E*)-1-(2-Methyl-4-phenyl­quinolin-3-yl)-3-(3-methyl­thio­phen-2-yl)prop-2-en-1-one

**DOI:** 10.1107/S1600536813004753

**Published:** 2013-02-23

**Authors:** R. Prasath, P. Bhavana, Seik Weng Ng, Edward R. T. Tiekink

**Affiliations:** a Department of Chemistry, BITS, Pilani–K. K. Birla Goa Campus, Goa 403 726, India; b Department of Chemistry, University of Malaya, 50603 Kuala Lumpur, Malaysia; c Chemistry Department, Faculty of Science, King Abdulaziz University, PO Box 80203 Jeddah, Saudi Arabia

## Abstract

In the title compound, C_24_H_19_NOS, the quinoline residue (r.m.s. deviation = 0.018 Å) is essentially orthogonal to both the phenyl [dihedral angle = 88.95 (8)°] and 2-thienyl [81.98 (9)°] rings. The carbonyl O atom lies to one side of the quinoline plane, the carbonyl C atom is almost coplanar and the remaining atoms of the chalcone residue lies to the other side, so that overall the mol­ecule has an L-shape. The conformation about the ethyl­ene bond [1.340 (2) Å] is *E*. In the crystal, a supra­molecular chain with the shape of a square rod aligned along the *b*-axis direction is sustained by C—H⋯π inter­actions, the π-systems being the heterocyclic rings.

## Related literature

For background details and the biological application of quinoline and quinoline chalcones, see: Joshi *et al.* (2011 ▶); Prasath & Bhavana (2012 ▶); Kalanithi *et al.* (2012 ▶); Prasath *et al.* (2013 ▶). For the structure of the dimethyl-substituted quinolinyl compound without a methyl substituent on the 2-thienyl ring, see: Prasath *et al.* (2011 ▶).
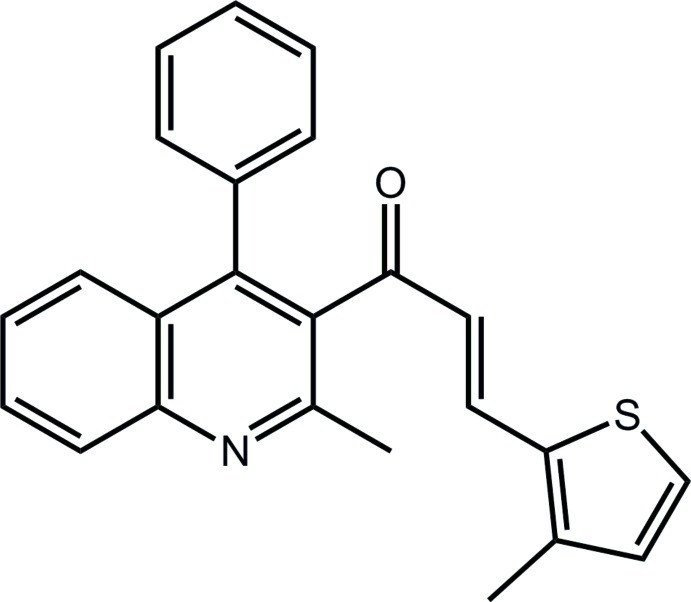


## Experimental

### 

#### Crystal data

C_24_H_19_NOS
*M* *_r_* = 369.46
Triclinic, 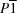

*a* = 10.0815 (7) Å
*b* = 10.2956 (7) Å
*c* = 10.5403 (7) Å
α = 71.013 (6)°
β = 78.697 (5)°
γ = 70.412 (6)°
*V* = 969.99 (11) Å^3^
*Z* = 2
Mo *K*α radiation
μ = 0.18 mm^−1^
*T* = 295 K
0.40 × 0.20 × 0.10 mm

#### Data collection

Agilent SuperNova Dual diffractometer with an Atlas detector
Absorption correction: multi-scan (*CrysAlis PRO*; Agilent, 2011 ▶) *T*_min_ = 0.780, *T*_max_ = 1.000
8430 measured reflections
4473 independent reflections
3484 reflections with *I* > 2σ(*I*)
*R*_int_ = 0.025

#### Refinement

*R*[*F* ^2^ > 2σ(*F* ^2^)] = 0.048
*wR*(*F* ^2^) = 0.135
*S* = 1.03
4473 reflections
246 parameters
H-atom parameters constrained
Δρ_max_ = 0.28 e Å^−3^
Δρ_min_ = −0.26 e Å^−3^

### 

Data collection: *CrysAlis PRO* (Agilent, 2011 ▶); cell refinement: *CrysAlis PRO*; data reduction: *CrysAlis PRO*; program(s) used to solve structure: *SHELXS97* (Sheldrick, 2008 ▶); program(s) used to refine structure: *SHELXL97* (Sheldrick, 2008 ▶); molecular graphics: *ORTEP-3 for Windows* (Farrugia, 2012 ▶) and *DIAMOND* (Brandenburg, 2006 ▶); software used to prepare material for publication: *publCIF* (Westrip, 2010 ▶).

## Supplementary Material

Crystal structure: contains datablock(s) global, I. DOI: 10.1107/S1600536813004753/hb7043sup1.cif

Structure factors: contains datablock(s) I. DOI: 10.1107/S1600536813004753/hb7043Isup2.hkl

Supplementary material file. DOI: 10.1107/S1600536813004753/hb7043Isup3.cml

Additional supplementary materials:  crystallographic information; 3D view; checkCIF report

## Figures and Tables

**Table 1:** Hydrogen-bond geometry (Å, °) *Cg*1 and *Cg*2 are the centroids of the S1,C20–C23 and N1,C1,C6–C9 rings, respectively.

*D*—H⋯*A*	*D*—H	H⋯*A*	*D*⋯*A*	*D*—H⋯*A*
C4—H4⋯*Cg*1^i^	0.93	2.88	3.688 (2)	146
C22—H22⋯*Cg*2^ii^	0.93	2.60	3.457 (2)	153

Symmetry codes: (i) 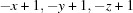; (ii) 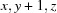.

**Fig. 1.**
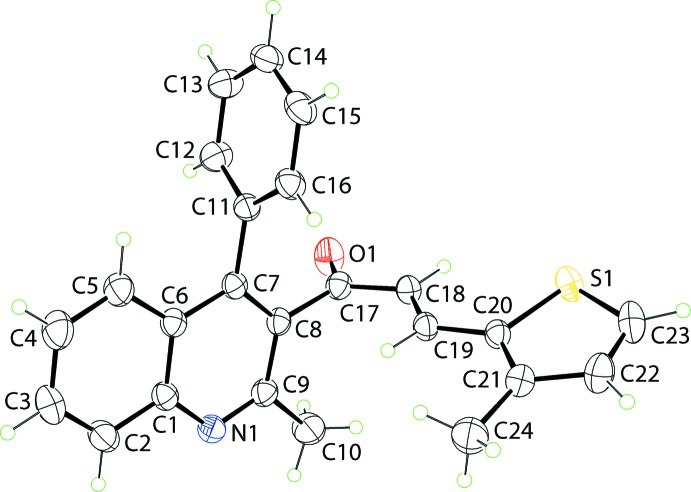
The molecular structure of (I) showing displacement ellipsoids at the 35% probability level.

**Fig. 2.**
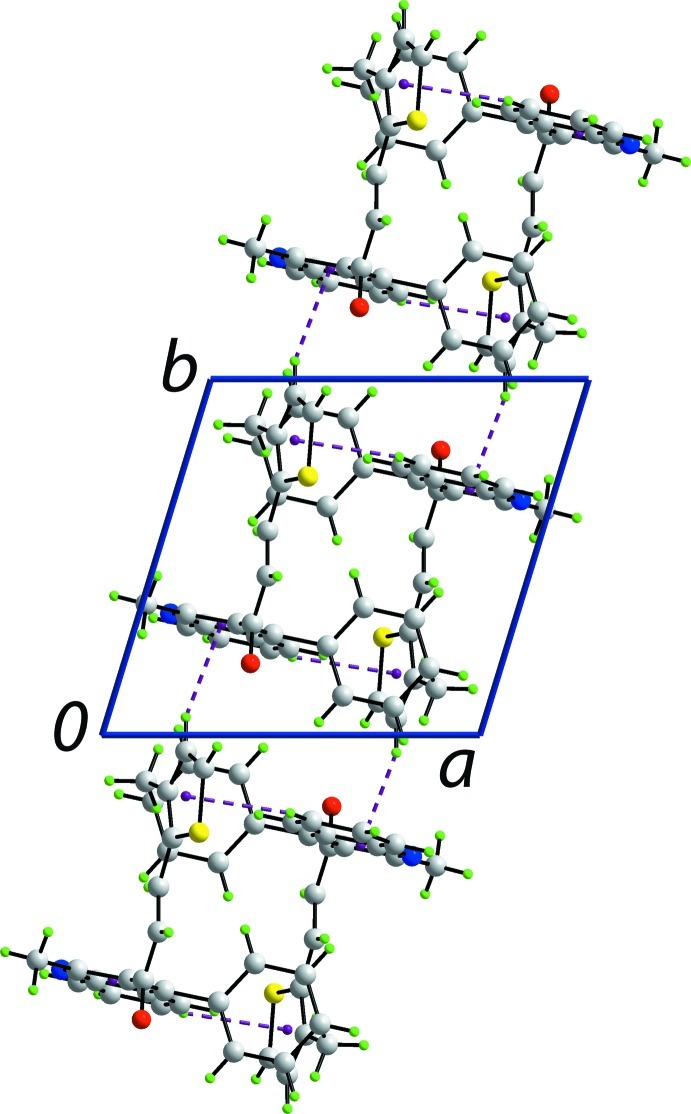
A view in projection down the c axis of the supramolecular chain in (I). The C—H···π interactions are shown as purple dashed lines.

**Fig. 3.**
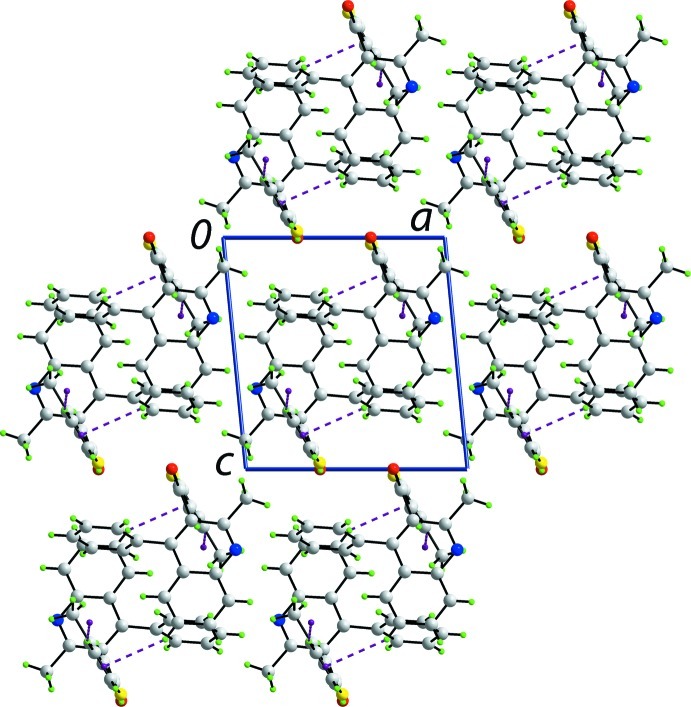
A view in projection down the b axis of the unit-cell content for (I). The C—H···π interactions are shown as purple dashed lines.

## supplementary crystallographic information

### Comment

In addition to their being valuable intermediates in organic synthesis (Prasath & Bhavana, 2012; Joshi *et al.*, 2011), quinoline and heterocyclic analogues, such as chalcones, exhibit a variety of biological activities, *e.g.* anti-plasmodial, anti-microbial and anticancer activities (Prasath *et al.*, 2013; Kalanithi *et al.*, 2012). The title quinolinyl/chalcone bearing a thienyl substituent, (I), was investigated in the context of the above.

In (I), Fig. 1, the phenyl ring is perpendicular to the quinolinyl residue (r.m.s. deviation = 0.018 Å), forming a dihedral angle of 88.95 (8)°. The 3-thienyl ring also occupies a position approximately orthogonal to the quinolinyl residue with a dihedral angle of 81.98 (9)°. With respect to the plane through the quinolinyl residue, the carbonyl-O1 atom lies to one side, the carbonyl-C17 atom is almost co-planar and the remaining chalcone residue lies to the other side so that the molecule has an *L*-shape. The conformation about the ethylene bond [1.340 (2) Å] is *E*. A similar conformation and displacement of atoms was found in the most closely related structure, namely that of the recently reported (2*E*)-1-(2,4-dimethylquinolin-3-yl)-3-(thiophen-2-yl)prop-2-en-1-one (Prasath *et al.*, 2011).

The most notable feature of the crystal packing is the formation of supramolecular chains along the *b* axis and sustained by C—H···π interactions between quinolinyl-C_6_—H4 and the 3-thienyl ring, and between 3-thienyl-H22 and the pyridyl ring, Fig. 2 and Table 1. Owing to the *L*-shape of the molecule, the chain has the shape of a square rod. Chains stack with no specific interactions between them, Fig. 3.

### Experimental

A mixture of 3-acetyl-2-methyl-4-phenylquinoline (1.3 g, 0.005 *M*), 3-methylthiophene-2-carbaldehyde (630 mg, 0.005 *M*) and KOH (0.5 g) in distilled ethanol (50 ml) was stirred for 12 h at room temperature. The resulting mixture was neutralized with dilute acetic acid. The deposited solid was filtered, dried and purified by column chromatography using a 1:1 mixture of ethyl acetate and hexane. Re-crystallization was by slow evaporation of an acetone solution of (I), which yielded colourless prisms in 76% yield; *M*.pt: 453–455 K.

### Refinement

The C-bound H atoms were geometrically placed (C—H = 0.95–0.96 Å) and refined as riding with *U_iso_*(H) = 1.2–1.5*U_eq_*(C).

### Crystal data

**Table:**

C_24_H_19_NOS	*Z* = 2
*M**_r_* = 369.46	*F*(000) = 388
Triclinic, *P*1	*D*_x_ = 1.265 Mg m^−^^3^
Hall symbol: -P 1	Mo *K*α radiation, λ = 0.71073 Å
*a* = 10.0815 (7) Å	Cell parameters from 2781 reflections
*b* = 10.2956 (7) Å	θ = 3.1–27.5°
*c* = 10.5403 (7) Å	µ = 0.18 mm^−^^1^
α = 71.013 (6)°	*T* = 295 K
β = 78.697 (5)°	Prism, colourless
γ = 70.412 (6)°	0.40 × 0.20 × 0.10 mm
*V* = 969.99 (11) Å^3^	

### Data collection

**Table:**

Agilent SuperNova Dual diffractometer with an Atlas detector	4473 independent reflections
Radiation source: SuperNova (Mo) X-ray Source	3484 reflections with *I* > 2σ(*I*)
Mirror monochromator	*R*_int_ = 0.025
Detector resolution: 10.4041 pixels mm^-1^	θ_max_ = 27.6°, θ_min_ = 3.1°
ω scan	*h* = −12→13
Absorption correction: multi-scan (*CrysAlis PRO*; Agilent, 2011)	*k* = −13→13
*T*_min_ = 0.780, *T*_max_ = 1.000	*l* = −12→13
8430 measured reflections	

### Refinement

**Table:**

Refinement on *F*^2^	Primary atom site location: structure-invariant direct methods
Least-squares matrix: full	Secondary atom site location: difference Fourier map
*R*[*F*^2^ > 2σ(*F*^2^)] = 0.048	Hydrogen site location: inferred from neighbouring sites
*wR*(*F*^2^) = 0.135	H-atom parameters constrained
*S* = 1.03	*w* = 1/[σ^2^(*F*_o_^2^) + (0.0573*P*)^2^ + 0.2365*P*] where *P* = (*F*_o_^2^ + 2*F*_c_^2^)/3
4473 reflections	(Δ/σ)_max_ < 0.001
246 parameters	Δρ_max_ = 0.28 e Å^−^^3^
0 restraints	Δρ_min_ = −0.26 e Å^−^^3^

### Special details

**Table:**

Geometry. All e.s.d.'s (except the e.s.d. in the dihedral angle between two l.s. planes) are estimated using the full covariance matrix. The cell e.s.d.'s are taken into account individually in the estimation of e.s.d.'s in distances, angles and torsion angles; correlations between e.s.d.'s in cell parameters are only used when they are defined by crystal symmetry. An approximate (isotropic) treatment of cell e.s.d.'s is used for estimating e.s.d.'s involving l.s. planes.
Refinement. Refinement of *F*^2^ against ALL reflections. The weighted *R*-factor *wR* and goodness of fit *S* are based on *F*^2^, conventional *R*-factors *R* are based on *F*, with *F* set to zero for negative *F*^2^. The threshold expression of *F*^2^ > σ(*F*^2^) is used only for calculating *R*-factors(gt) *etc*. and is not relevant to the choice of reflections for refinement. *R*-factors based on *F*^2^ are statistically about twice as large as those based on *F*, and *R*- factors based on ALL data will be even larger.

### Fractional atomic coordinates and isotropic or equivalent isotropic displacement parameters (Å^2^)

**Table:**

	*x*	*y*	*z*	*U*_iso_*/*U*_eq_	
S1	0.33532 (6)	0.72592 (6)	0.97283 (5)	0.05153 (17)	
O1	0.33453 (18)	0.20079 (15)	1.00072 (14)	0.0629 (4)	
N1	0.08222 (15)	0.34043 (16)	0.65204 (16)	0.0454 (4)	
C1	0.18240 (18)	0.31032 (18)	0.54916 (17)	0.0399 (4)	
C2	0.1367 (2)	0.3073 (2)	0.4323 (2)	0.0527 (5)	
H2	0.0406	0.3285	0.4262	0.063*	
C3	0.2316 (3)	0.2738 (2)	0.3286 (2)	0.0602 (5)	
H3	0.2002	0.2718	0.2522	0.072*	
C4	0.3757 (2)	0.2425 (3)	0.3361 (2)	0.0608 (6)	
H4	0.4397	0.2196	0.2645	0.073*	
C5	0.4241 (2)	0.2449 (2)	0.44696 (19)	0.0511 (5)	
H5	0.5208	0.2241	0.4502	0.061*	
C6	0.32869 (18)	0.27881 (17)	0.55698 (17)	0.0379 (4)	
C7	0.37112 (17)	0.28190 (17)	0.67702 (17)	0.0363 (4)	
C8	0.26889 (17)	0.31240 (17)	0.77857 (17)	0.0367 (4)	
C9	0.12435 (18)	0.33970 (19)	0.76224 (19)	0.0432 (4)	
C10	0.0110 (2)	0.3675 (3)	0.8747 (2)	0.0672 (6)	
H10A	−0.0802	0.3905	0.8445	0.101*	
H10B	0.0164	0.4466	0.9008	0.101*	
H10C	0.0244	0.2833	0.9505	0.101*	
C11	0.52458 (17)	0.24958 (18)	0.69071 (16)	0.0377 (4)	
C12	0.6053 (2)	0.1100 (2)	0.7428 (2)	0.0514 (5)	
H12	0.5634	0.0361	0.7717	0.062*	
C13	0.7480 (2)	0.0793 (2)	0.7525 (2)	0.0561 (5)	
H13	0.8016	−0.0151	0.7874	0.067*	
C14	0.8112 (2)	0.1878 (2)	0.71062 (19)	0.0520 (5)	
H14	0.9074	0.1666	0.7162	0.062*	
C15	0.7321 (2)	0.3266 (2)	0.6608 (2)	0.0526 (5)	
H15	0.7745	0.4002	0.6334	0.063*	
C16	0.5892 (2)	0.3582 (2)	0.65095 (19)	0.0467 (4)	
H16	0.5360	0.4531	0.6174	0.056*	
C17	0.30768 (19)	0.31202 (19)	0.90990 (18)	0.0415 (4)	
C18	0.30830 (19)	0.44686 (19)	0.92575 (18)	0.0416 (4)	
H18	0.3353	0.4453	1.0059	0.050*	
C19	0.27227 (18)	0.57334 (18)	0.83162 (17)	0.0391 (4)	
H19	0.2463	0.5721	0.7522	0.047*	
C20	0.26953 (18)	0.71087 (18)	0.84036 (17)	0.0385 (4)	
C21	0.2191 (2)	0.84246 (19)	0.75107 (18)	0.0454 (4)	
C22	0.2355 (2)	0.9534 (2)	0.7922 (2)	0.0590 (5)	
H22	0.2071	1.0497	0.7435	0.071*	
C23	0.2960 (3)	0.9070 (2)	0.9081 (2)	0.0606 (6)	
H23	0.3141	0.9670	0.9480	0.073*	
C24	0.1539 (3)	0.8692 (3)	0.6258 (2)	0.0666 (6)	
H24A	0.2032	0.7936	0.5843	0.100*	
H24B	0.1604	0.9595	0.5644	0.100*	
H24C	0.0563	0.8715	0.6484	0.100*	

### Atomic displacement parameters (Å^2^)

**Table:**

	*U*^11^	*U*^22^	*U*^33^	*U*^12^	*U*^13^	*U*^23^
S1	0.0653 (3)	0.0586 (3)	0.0420 (3)	−0.0270 (3)	−0.0072 (2)	−0.0187 (2)
O1	0.0963 (12)	0.0451 (8)	0.0471 (8)	−0.0234 (8)	−0.0149 (8)	−0.0052 (6)
N1	0.0385 (8)	0.0491 (9)	0.0537 (9)	−0.0144 (7)	−0.0028 (7)	−0.0203 (7)
C1	0.0421 (9)	0.0381 (9)	0.0429 (9)	−0.0148 (7)	−0.0045 (7)	−0.0126 (7)
C2	0.0524 (11)	0.0573 (12)	0.0557 (11)	−0.0191 (10)	−0.0130 (9)	−0.0178 (10)
C3	0.0766 (15)	0.0704 (14)	0.0451 (11)	−0.0282 (12)	−0.0101 (10)	−0.0222 (10)
C4	0.0661 (14)	0.0750 (15)	0.0488 (11)	−0.0261 (12)	0.0074 (10)	−0.0290 (11)
C5	0.0475 (10)	0.0645 (12)	0.0479 (10)	−0.0219 (10)	0.0050 (9)	−0.0245 (9)
C6	0.0401 (9)	0.0364 (8)	0.0404 (9)	−0.0156 (7)	−0.0011 (7)	−0.0120 (7)
C7	0.0380 (8)	0.0321 (8)	0.0412 (9)	−0.0137 (7)	−0.0025 (7)	−0.0108 (7)
C8	0.0396 (9)	0.0345 (8)	0.0392 (8)	−0.0135 (7)	−0.0007 (7)	−0.0135 (7)
C9	0.0381 (9)	0.0454 (10)	0.0493 (10)	−0.0135 (8)	0.0028 (8)	−0.0199 (8)
C10	0.0439 (11)	0.1004 (18)	0.0656 (14)	−0.0208 (12)	0.0099 (10)	−0.0432 (13)
C11	0.0366 (9)	0.0420 (9)	0.0371 (8)	−0.0139 (7)	−0.0011 (7)	−0.0134 (7)
C12	0.0469 (10)	0.0448 (10)	0.0613 (12)	−0.0176 (9)	−0.0012 (9)	−0.0114 (9)
C13	0.0451 (11)	0.0522 (12)	0.0639 (13)	−0.0066 (9)	−0.0087 (9)	−0.0128 (10)
C14	0.0379 (9)	0.0735 (14)	0.0508 (11)	−0.0182 (10)	−0.0040 (8)	−0.0239 (10)
C15	0.0509 (11)	0.0640 (13)	0.0551 (11)	−0.0318 (10)	−0.0013 (9)	−0.0194 (10)
C16	0.0476 (10)	0.0449 (10)	0.0523 (11)	−0.0189 (8)	−0.0074 (8)	−0.0129 (8)
C17	0.0421 (9)	0.0416 (9)	0.0419 (9)	−0.0136 (8)	0.0001 (7)	−0.0142 (8)
C18	0.0449 (9)	0.0446 (10)	0.0395 (9)	−0.0130 (8)	−0.0047 (7)	−0.0171 (8)
C19	0.0414 (9)	0.0427 (9)	0.0380 (9)	−0.0134 (8)	−0.0026 (7)	−0.0172 (7)
C20	0.0397 (9)	0.0426 (9)	0.0374 (8)	−0.0139 (7)	0.0003 (7)	−0.0170 (7)
C21	0.0489 (10)	0.0430 (10)	0.0456 (10)	−0.0133 (8)	−0.0019 (8)	−0.0157 (8)
C22	0.0767 (14)	0.0408 (10)	0.0626 (13)	−0.0196 (10)	−0.0046 (11)	−0.0173 (9)
C23	0.0802 (15)	0.0572 (12)	0.0616 (13)	−0.0354 (12)	0.0030 (11)	−0.0293 (11)
C24	0.0771 (15)	0.0589 (13)	0.0609 (13)	−0.0090 (12)	−0.0250 (12)	−0.0138 (11)

### Geometric parameters (Å, º)

**Table:**

S1—C23	1.698 (2)	C11—C16	1.389 (2)
S1—C20	1.7281 (17)	C12—C13	1.382 (3)
O1—C17	1.217 (2)	C12—H12	0.9300
N1—C9	1.310 (2)	C13—C14	1.376 (3)
N1—C1	1.368 (2)	C13—H13	0.9300
C1—C6	1.413 (2)	C14—C15	1.366 (3)
C1—C2	1.410 (3)	C14—H14	0.9300
C2—C3	1.358 (3)	C15—C16	1.384 (3)
C2—H2	0.9300	C15—H15	0.9300
C3—C4	1.390 (3)	C16—H16	0.9300
C3—H3	0.9300	C17—C18	1.453 (2)
C4—C5	1.362 (3)	C18—C19	1.340 (2)
C4—H4	0.9300	C18—H18	0.9300
C5—C6	1.412 (2)	C19—C20	1.439 (2)
C5—H5	0.9300	C19—H19	0.9300
C6—C7	1.426 (2)	C20—C21	1.370 (3)
C7—C8	1.370 (2)	C21—C22	1.413 (3)
C7—C11	1.495 (2)	C21—C24	1.494 (3)
C8—C9	1.423 (2)	C22—C23	1.345 (3)
C8—C17	1.509 (2)	C22—H22	0.9300
C9—C10	1.505 (2)	C23—H23	0.9300
C10—H10A	0.9600	C24—H24A	0.9600
C10—H10B	0.9600	C24—H24B	0.9600
C10—H10C	0.9600	C24—H24C	0.9600
C11—C12	1.381 (3)		
			
C23—S1—C20	91.61 (10)	C13—C12—H12	119.7
C9—N1—C1	118.32 (15)	C14—C13—C12	120.29 (19)
N1—C1—C6	122.78 (16)	C14—C13—H13	119.9
N1—C1—C2	118.05 (16)	C12—C13—H13	119.9
C6—C1—C2	119.15 (16)	C15—C14—C13	119.83 (18)
C3—C2—C1	120.68 (19)	C15—C14—H14	120.1
C3—C2—H2	119.7	C13—C14—H14	120.1
C1—C2—H2	119.7	C14—C15—C16	120.21 (17)
C2—C3—C4	120.30 (19)	C14—C15—H15	119.9
C2—C3—H3	119.9	C16—C15—H15	119.9
C4—C3—H3	119.9	C15—C16—C11	120.58 (18)
C5—C4—C3	120.85 (19)	C15—C16—H16	119.7
C5—C4—H4	119.6	C11—C16—H16	119.7
C3—C4—H4	119.6	O1—C17—C18	121.50 (17)
C4—C5—C6	120.50 (18)	O1—C17—C8	119.87 (15)
C4—C5—H5	119.8	C18—C17—C8	118.60 (15)
C6—C5—H5	119.8	C19—C18—C17	123.79 (16)
C1—C6—C5	118.52 (16)	C19—C18—H18	118.1
C1—C6—C7	117.62 (15)	C17—C18—H18	118.1
C5—C6—C7	123.85 (16)	C18—C19—C20	126.93 (16)
C8—C7—C6	118.51 (15)	C18—C19—H19	116.5
C8—C7—C11	121.58 (15)	C20—C19—H19	116.5
C6—C7—C11	119.90 (14)	C21—C20—C19	127.31 (16)
C7—C8—C9	119.67 (16)	C21—C20—S1	111.30 (13)
C7—C8—C17	120.94 (15)	C19—C20—S1	121.39 (13)
C9—C8—C17	119.35 (15)	C20—C21—C22	111.32 (18)
N1—C9—C8	123.07 (15)	C20—C21—C24	125.56 (17)
N1—C9—C10	116.42 (16)	C22—C21—C24	123.12 (18)
C8—C9—C10	120.50 (17)	C23—C22—C21	113.90 (19)
C9—C10—H10A	109.5	C23—C22—H22	123.1
C9—C10—H10B	109.5	C21—C22—H22	123.1
H10A—C10—H10B	109.5	C22—C23—S1	111.86 (15)
C9—C10—H10C	109.5	C22—C23—H23	124.1
H10A—C10—H10C	109.5	S1—C23—H23	124.1
H10B—C10—H10C	109.5	C21—C24—H24A	109.5
C12—C11—C16	118.55 (16)	C21—C24—H24B	109.5
C12—C11—C7	120.36 (15)	H24A—C24—H24B	109.5
C16—C11—C7	121.08 (16)	C21—C24—H24C	109.5
C11—C12—C13	120.52 (17)	H24A—C24—H24C	109.5
C11—C12—H12	119.7	H24B—C24—H24C	109.5
			
C9—N1—C1—C6	−0.1 (3)	C8—C7—C11—C16	−90.2 (2)
C9—N1—C1—C2	178.26 (16)	C6—C7—C11—C16	91.0 (2)
N1—C1—C2—C3	−178.02 (18)	C16—C11—C12—C13	−1.3 (3)
C6—C1—C2—C3	0.4 (3)	C7—C11—C12—C13	178.36 (18)
C1—C2—C3—C4	−0.3 (3)	C11—C12—C13—C14	0.3 (3)
C2—C3—C4—C5	−0.1 (3)	C12—C13—C14—C15	0.8 (3)
C3—C4—C5—C6	0.3 (3)	C13—C14—C15—C16	−0.7 (3)
N1—C1—C6—C5	178.14 (16)	C14—C15—C16—C11	−0.3 (3)
C2—C1—C6—C5	−0.2 (2)	C12—C11—C16—C15	1.3 (3)
N1—C1—C6—C7	−1.3 (2)	C7—C11—C16—C15	−178.31 (17)
C2—C1—C6—C7	−179.58 (15)	C7—C8—C17—O1	−87.5 (2)
C4—C5—C6—C1	−0.1 (3)	C9—C8—C17—O1	90.2 (2)
C4—C5—C6—C7	179.22 (17)	C7—C8—C17—C18	94.1 (2)
C1—C6—C7—C8	1.2 (2)	C9—C8—C17—C18	−88.2 (2)
C5—C6—C7—C8	−178.19 (16)	O1—C17—C18—C19	−176.33 (18)
C1—C6—C7—C11	−179.93 (15)	C8—C17—C18—C19	2.0 (3)
C5—C6—C7—C11	0.7 (2)	C17—C18—C19—C20	179.62 (16)
C6—C7—C8—C9	0.1 (2)	C18—C19—C20—C21	−172.65 (18)
C11—C7—C8—C9	−178.75 (15)	C18—C19—C20—S1	7.9 (3)
C6—C7—C8—C17	177.80 (14)	C23—S1—C20—C21	−0.25 (14)
C11—C7—C8—C17	−1.1 (2)	C23—S1—C20—C19	179.28 (15)
C1—N1—C9—C8	1.5 (3)	C19—C20—C21—C22	−179.31 (17)
C1—N1—C9—C10	−177.62 (17)	S1—C20—C21—C22	0.2 (2)
C7—C8—C9—N1	−1.6 (3)	C19—C20—C21—C24	1.0 (3)
C17—C8—C9—N1	−179.27 (15)	S1—C20—C21—C24	−179.51 (16)
C7—C8—C9—C10	177.54 (18)	C20—C21—C22—C23	0.0 (3)
C17—C8—C9—C10	−0.2 (3)	C24—C21—C22—C23	179.70 (19)
C8—C7—C11—C12	90.2 (2)	C21—C22—C23—S1	−0.2 (3)
C6—C7—C11—C12	−88.7 (2)	C20—S1—C23—C22	0.24 (17)

### Hydrogen-bond geometry (Å, º)

Cg1 and Cg2 are the centroids of the S1,C20–C23 and N1,C1,C6–C9 rings, respectively.

**Table:**

*D*—H···*A*	*D*—H	H···*A*	*D*···*A*	*D*—H···*A*
C4—H4···*Cg*1^i^	0.93	2.88	3.688 (2)	146
C22—H22···*Cg*2^ii^	0.93	2.60	3.457 (2)	153

Symmetry codes: (i) −*x*+1, −*y*+1, −*z*+1; (ii) *x*, *y*+1, *z*.
